# Testing a computerised tool to improve physical health monitoring in a medium secure forensic setting

**DOI:** 10.1192/bjo.2021.593

**Published:** 2021-06-18

**Authors:** Mike Smith, Mustafa Abbas

**Affiliations:** West London NHS Trust

## Abstract

**Aims:**

This project aimed to improve adherence to regular monitoring of the physical health of inpatients within a medium secure forensic psychiatric unit. A computerised tool to remind doctors to do checks was created, which was proposed would improve adherence.

**Background:**

The physical health of people with mental health problems is of some concern, with higher rates of physical comorbidity and mortality compared to the general population.

The forensic inpatient population has a high burden of both severe mental illness and physical ill health, and a high medication burden with potential adverse effects on physical health.

To support the health of patients in our medium secure unit, each should routinely have three physical health checks done at least every six months. These are 1) an electrocardiogram (ECG), 2) a set of blood tests and 3) a full physical examination.

**Method:**

Patient records for 26 patients across two medium secure psychiatric wards were checked for 1) an ECG, 2) a full set of blood tests and 3) a full general physical examination within the past 6 months.

A tool was created that automatically calculated the next due date for each check and colour coded which were overdue (red) or within 30 days of the due date (yellow). This tool was given to the core trainees working on these wards to help them keep track of which checks needed to be done.

The records for patients on the same two wards were rechecked four months later and the adherence rates compared.

**Result:**

On both wards, for each of the three physical health checks, a substantial improvement was seen in the proportion completed within the past 6 months.

**Conclusion:**

The tool created was a useful means of presenting, in one place, relevant information needed by doctors working in medium secure forensic wards regarding physical health checks, and drawing their attention to tasks that needed to be done. This led to an improvement in the adherence to physical health monitoring in these wards. An area for future improvement was identified regarding the unit's capacity to perform ECGs in a timely manner.

